# Jacob’s Disease: Case Series, Extensive Literature Review and Classification Proposal

**DOI:** 10.3390/jcm12030938

**Published:** 2023-01-25

**Authors:** Luca Raccampo, Giorgio Panozzo, Alessandro Tel, Michele Di Cosola, Gianluca Colapinto, Lorenzo Trevisiol, Antonio D’agostino, Salvatore Sembronio, Massimo Robiony

**Affiliations:** 1 Maxillofacial Surgery Department, Academic Hospital of Udine, Department of Medicine, University of Udine, 33100 Udine, Italy; 2 Section of Oral and Maxillofacial Surgery, Department of Surgical Sciences, Dentistry, Gynaecology and Paediatrics, University of Verona, 37129 Verona, Italy; 3 Section of Dentistry and Dental Prosthetics, Department of Medicine, University of Foggia, 71122 Foggia, Italy; 4 Independent Researcher, 70121 Bari, Italy

**Keywords:** Jacob’s disease, coronoid hyperplasia, osteochondroma, temporomandibular surgery

## Abstract

Jacob’s disease is a rare entity consisting of the formation of a pseudojoint between an abnormal coronoid process of the mandible and the inner surface of the zygomatic bone. First described by Jacob in 1899, its diagnosis and definition have never been entirely univocal. In this paper, we present three emblematic cases and an extensive review of the literature on Jacob’s disease. Given the variability observed in the presentation of the disease, we have developed a proposal for the classification, here reported.

## 1. Introduction

In 1899, Oscar Jacob first described the eponymous disease. He noticed, as a post-mortem relief, a restricted jaw range of motion in a patient caused by an impingement between the inner side of the zygoma and the coronoid process of the mandible (CPM) [1]. He observed that the two bony surfaces were joined by bands of fibrous tissue, mimicking a new joint. Nearly 50 years earlier, in 1853, Langenbeck described the first case of an enlargement of the CPM [2]. Subsequently, several cases of Jacob’s disease (JD) have been reported in the literature but without a clear and unambiguous definition of the condition ever being given. This confusion over the classification of the different forms of coronoid enlargement and JD is partly due to the misuse of the terms osteoma (OS), cartilage-capped exostosis (CCE) and osteochondroma (OC) and the latter’s apparent link with JD [3]. Differences in the proportion of cartilage and bone elements in the sample justified different histological diagnoses. All these pathological entities, as well as true coronoid hyperplasia (CH), can determine an enlargement of the coronoid and potentially lead to JD. While, for some authors, the presence of an osteochondroma of the CPM must be a prerequisite for diagnosing JD [4,5,6,7]; this is not a defined and agreed point. In fact, it is defined by most as the formation of a pseudojoint between the inner surface of the zygoma and the CPM, which can be deformed or elongated by several pathological processes [8,9,10,11,12,13,14]. The authors report here three cases of JD that express well the broad spectrum of the pathology, as well as an extensive and meticulous literature review, aiming to propose a possible clinical and radiological classification of this disease.

## 2. Review of the Literature

After having established the definition of JD as a condition where a pseudojoint forms between an abnormal coronoid process and the inner aspect of the zygoma, with a broad spectrum of presentation and pathology, we conducted a comprehensive literature review to identify the cases that matched this definition or were already identified as JD by the authors. Articles were retrieved from PubMed, Cochrane and Semantic scholar databases using the following search terms: “Jacob’s disease”, “Coronoid Osteochondroma”, “Coronoid hyperplasia”, “Coronoid process pseudojoint” and “Coronoid process enlargement”. Articles were also retrieved indirectly by screening the references of papers identified through the aforementioned keywords. The results were filtered, limiting the selection to papers written in the English language. Cases in which a certain JD diagnosis could not be established through author declaration, imaging or inferences were excluded. Full texts of all articles meeting the inclusion criteria were thoroughly reviewed for data extraction. We extracted reports of age, gender, the side and type of the CPM anomaly, MMO before and after surgery, surgical treatment approach, follow-up period and reported recurrence. A total of 116 cases, including the 3 cases hereafter reported, were selected from a total of 107 articles, and the data collected are summarized in Table 1 [1,4,6,7,8,11,12,13,14,15,16,17,18,19,20,21,22,23,24,25,26,27,28,29,30,31,32,33,34,35,36,37,38,39,40,41,42,43,44,45,46,47,48,49,50,51,52,53,54,55,56,57,58,59,60,61,62,63,64,65,66,67,68,69,70,71,72,73,74,75,76,77,78,79,80,81,82,83,84,85,86,87,88,89,90,91,92,93,94,95,96,97,98,99,100,101,102,103,104,105,106,107,108,109,110,111].

## 3. Case Presentation

### 3.1. Case 1

**Figure 1:**
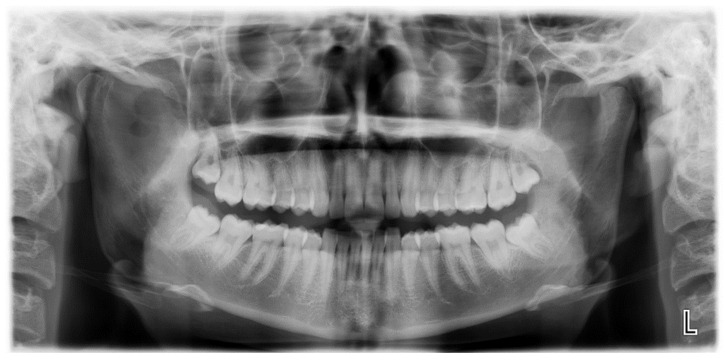
Patient’s OPG showing no evident anomalies.

**Figure 2:**
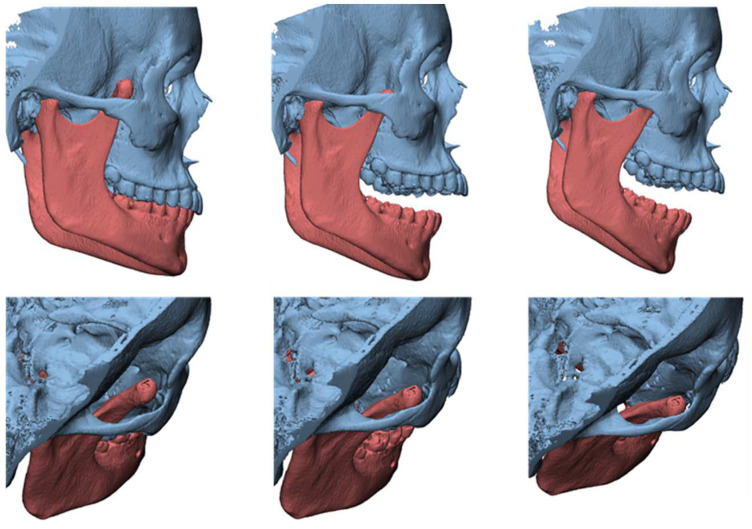
Cinematic 3D reconstruction (Mimics, Materialise NV, Leuven, Belgium) showing the pseudojoint established between the altered right CPM and the inner aspect of zygomatic bone.

**Figure 3:**
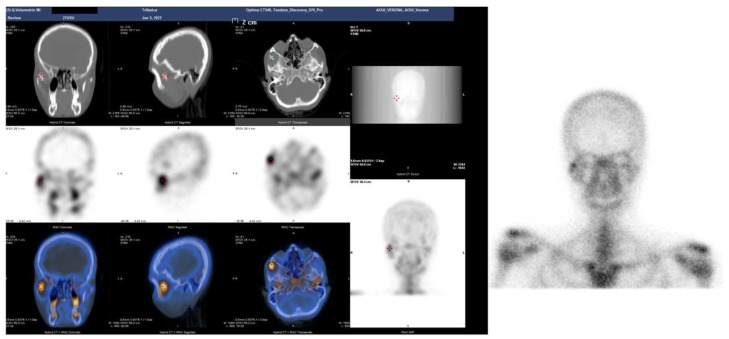
SPET-CT showing bone reworking in correspondence to the joint between the jaw and the right zygomatic bone compared to the contralateral homologous site.

**Figure 4:**
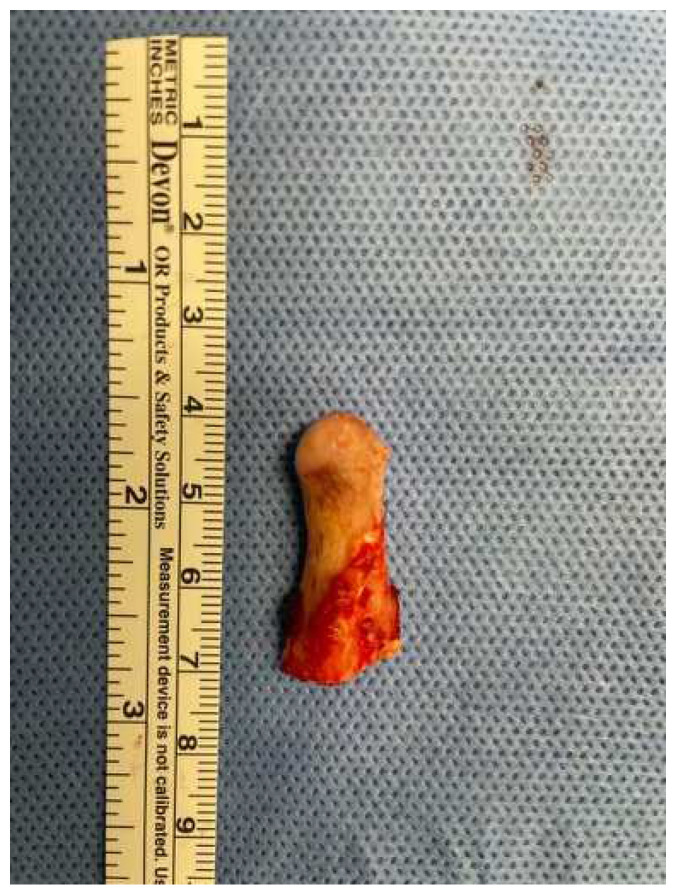
Right mushroom-shaped coronoid process excised.

A 19-year-old woman was referred to us, presenting with a history of progressive limitation in mouth opening over the previous 18 months. She complained of a dull pain just in front of her right ear, exacerbated by palpation and mandibular movements. She had no medical history and did not report any previous local trauma. She was initially diagnosed with temporomandibular disease (TMD) dysfunction and treated conservatively with an occlusal bite with no symptomatologic relief. At physical examination, the maximum mouth opening (MMO) was reduced to 25 mm, but mandibular movements were preserved. No articular noises during temporomandibular joint (TMJ) bilateral palpation were perceived, but she complained of pain in the masticatory muscles bilaterally. No malocclusion, facial asymmetry or swelling were identified. The orthopantomogram (OPG) (Figure 1) and the magnetic resonance imaging (MRI) were negative. Given the absence of clinical improvement but a rather slow and progressive reduction in MMO, a computed tomography (CT) was performed, revealing an enlargement and an abnormally shaped right CPM, establishing a close relationship with the zygomatic arch (Figure 2). Furthermore, single-photon emission computed tomography (SPECT) was prescribed, and the late images of bone uptake showed a focal increase in the correspondence of the so-called pseudojoint between the jaw and the right zygomatic bone compared to the left side (Figure 3). According to this, a provisional diagnosis of JD was made. The patient underwent a right coronoidectomy through an intraoral approach under general anesthesia with awake, blind, nasal intubation. The right, mushroom-shaped coronoid process was identified and resected, and a sort of fibrotic capsule between the inner aspect of the zygoma and the CPM was highlighted, establishing the pseudojoint, with this confirming the diagnosis of JD (Figure 4). Histopathologically, the specimen showed a growth pattern of hyperplastic bone tissue covered by cartilaginous layers and an external coating of dense fibrous tissue. There were no intra-operative or immediate postoperative complications. Postoperative CT was obtained for baseline follow-up. The MMO increased to about 30 mm in the immediate postoperative period, and this improved to 40 mm after a month of aggressive physiotherapy. She underwent clinical and radiological follow-up, and after 12 months, the MMO was stable, and no recurrence was observed.

### 3.2. Case 2

**Figure 5:**
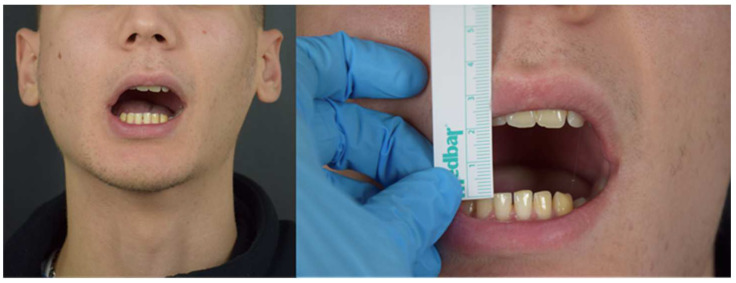
Patient showing an MMO of 20 mm and a slight deviation to the right at first physical examination.

**Figure 6:**
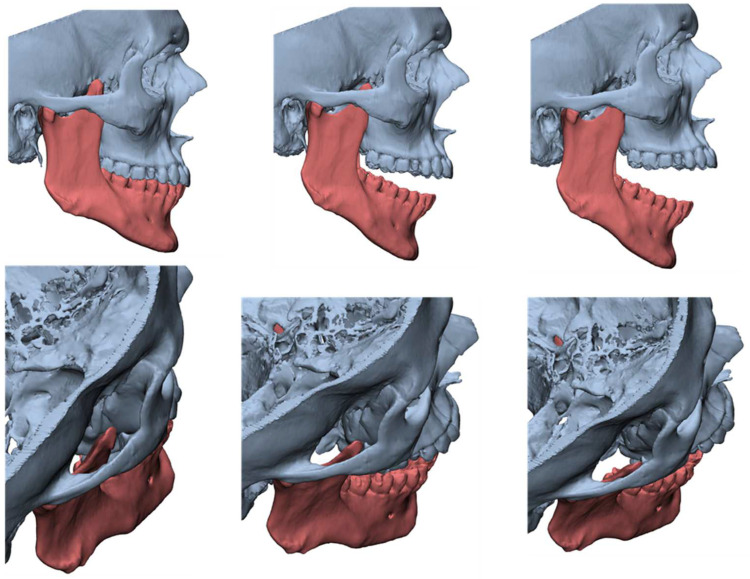
Cinematic 3D reconstruction (Mimics, Materialise NV., Leuven, Belgium) showing the established right JD.

**Figure 7:**
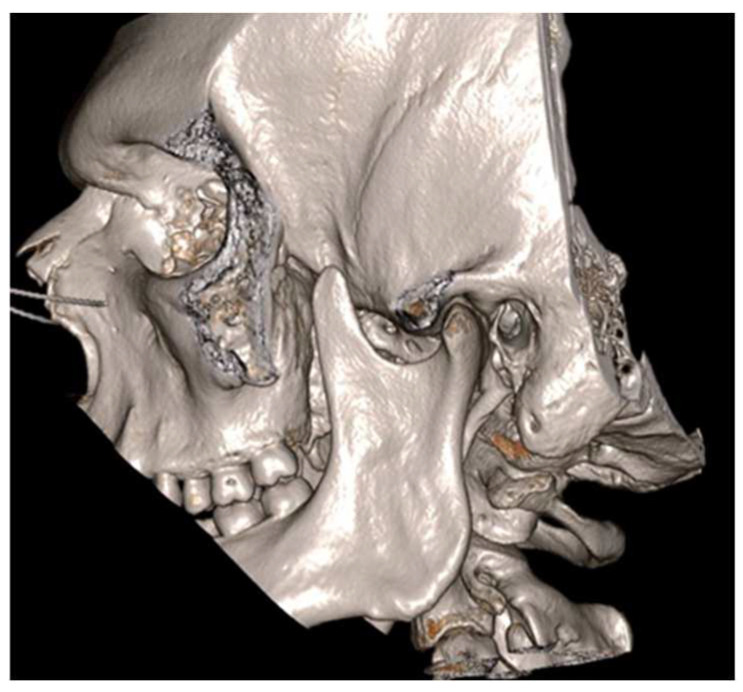
3D CT reconstructions showing an enlarged left CPM.

**Figure 8:**
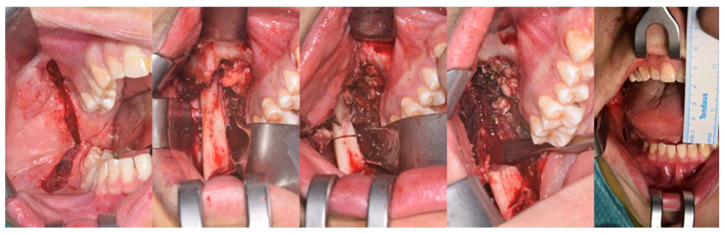
Surgical sequence of the intraoral coronoidectomy. The patient shows an immediate improvement in the MMO up to 40 mm.

**Figure 9:**
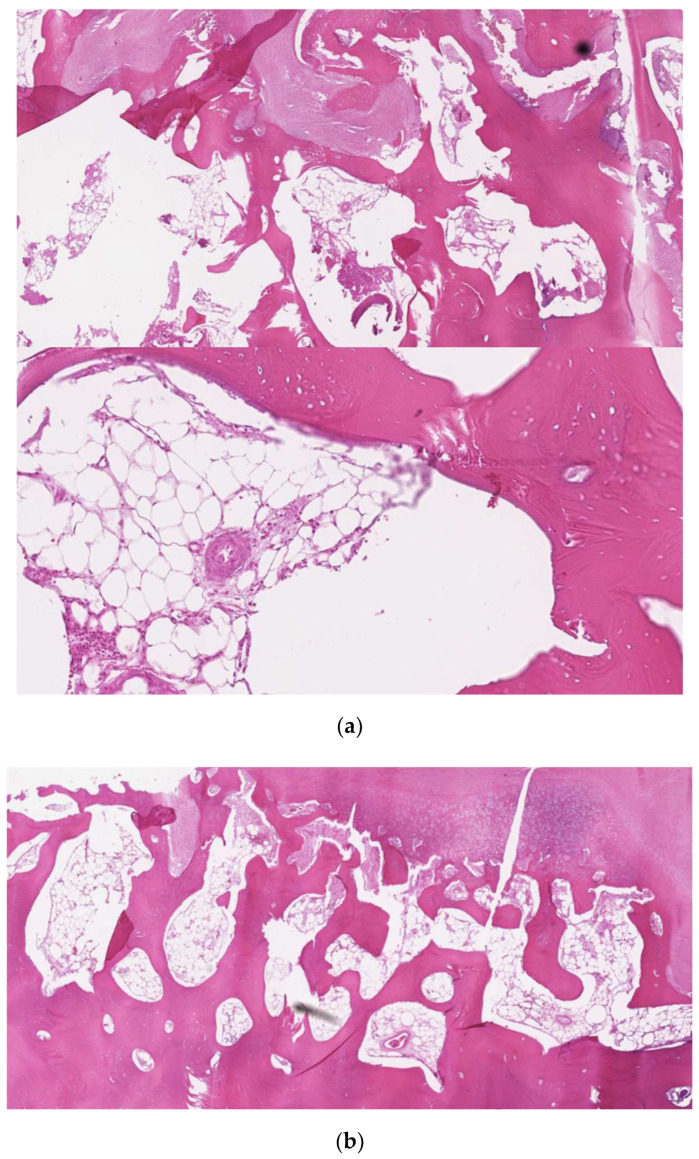
Hematoxylin–eosin staining of the lesion. Sections of compact, trabecular bone tissue covered by a layer of hyaline cartilage and fibrous cartilage overlaid by dense fibrous connective tissue (**a**,**b**).

An 18-year-old male patient with a 24-month history of worsening reductions in mouth opening was referred to our attention for an evaluation of suspected TMD. No previous history of trauma was reported by the patient, and the medical history did not report any relevant elements. A physical examination showed an MMO of 20 mm with a deviation to the right during mouth opening (Figure 5). The patient did not complain of pain at the palpation of TMJ bilaterally. The masticatory muscles were painful bilaterally and especially on the right side. No TMJ clicking or crepitus were detectable. The endfeel was rigid and painful on the right side. At the OPG, the right glenoid fossa and condyle were not totally distinguishable, so an MRI and CT scan were requested to examine the case in depth. The MRI was negative for TMD, but in the CT scan, an enlarged right CPM with a mushroom-shaped end was highlighted, establishing a close relationship with the inner aspect of the right zygoma, which seemed impressed by the CPM forming a pseudojoint (Figure 6). It is interesting to notice how also the contralateral CPM was slightly elongated (Figure 7). The suspected diagnosis was of a right JD. The patient was subsequently submitted to an intraoral right coronoidectomy under general anesthesia. An incision along the vestibular aspect of the ascending mandibular ramus was performed, then a sub-periosteal dissection exposing the right maxillary zygomatic arch and the anterior rim of the mandibular ascending ramus was executed. At this point, the CPM was detected as showing a protuberance covered with fibrous tissue mimicking a capsule. Immediately after the completion of the coronoidectomy, the MMO increased to 40 mm (Figure 8). The histopathology of the CPM showed sections of compact, trabecular bone tissue covered by a layer of hyaline cartilage and fibrous cartilage overlaid by dense fibrous connective tissue—compatible with the diagnosis of osteochondroma (Figure 9). At 1 week, postoperative jaw-opening exercises and articular physiotherapy with TheraBite jaw motion rehabilitation system ^TM^ (Atos medical, Padua, Italy) were prescribed. At 1 month follow-up, the patient had an MMO of 20 mm, 18 mm at 2 months and 10 mm at 3 months. The cause of this negative trend was initially identified as the fibrotic retraction of the intraoral scar of the surgical access. At 5 months follow-up, the MMO was 32 mm and increased to 38 mm 1 month later. At 12 months follow-up, the MMO was 44 mm. A control CT scan was conducted and showed no recurrence and no significant changes on the contralateral side.

### 3.3. Case 3

**Figure 10:**
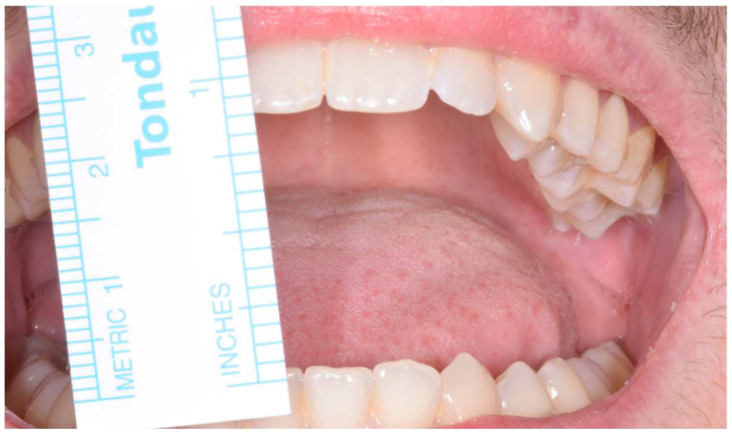
MMO of 20 mm at first clinical examination.

**Figure 11:**
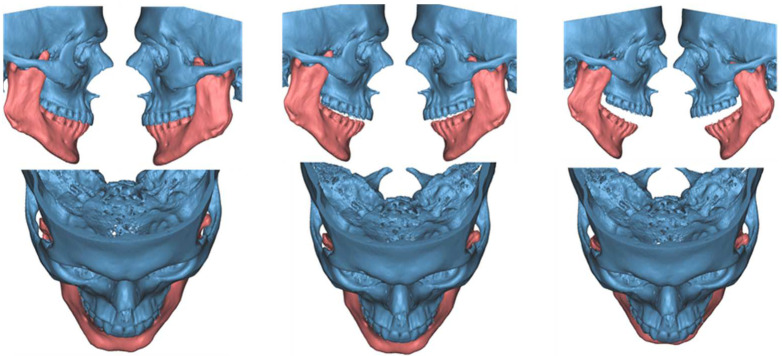
3-dimensional dynamic reconstruction showing the bilateral pseudojoint determining a bilateral JD.

**Figure 12:**
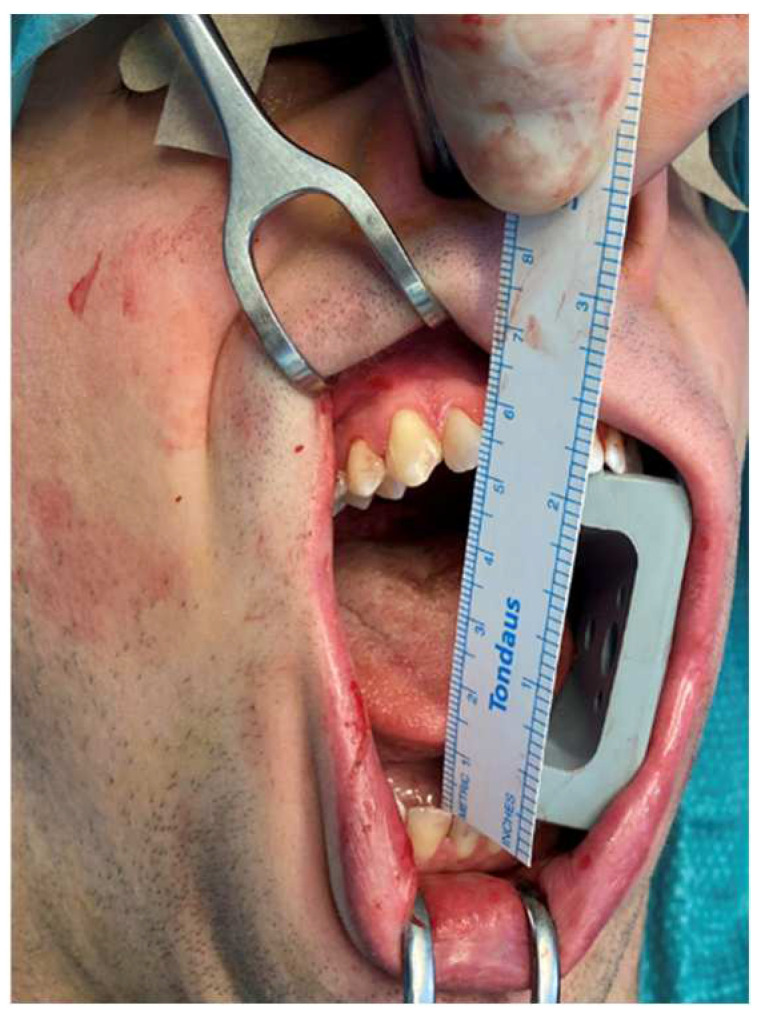
MMO of 50 mm right after bilateral coronoidectomy.

A 23-year-old male patient presented complaining of a progressive limitation in mouth opening. He did not report any pain. The reduction in the MMO had been worsening over the prior 5 years. There was no history of trauma. Clinical examination revealed an MMO of 20 mm with no deviation (Figure 10). There was no pain during jaw opening. He did not experience pain on palpation of the preauricular area bilaterally. The endfeel was rigid but not painful, and crepitus was perceived at the right TMJ. OPG and TMJ projections showed a lack of translation of the mandibular condyles in the opening and an alteration in the morphology of the CPM bilaterally. MRI was negative for intra-articular causes of ankylosis. CT scans showed a bilateral enlargement of the CPMs with a mushroom-shaped bony outgrowth forming a pseudojoint with the inner aspect of the zygomatic bones, which appeared modeled to the CPMs. This is particularly evident in the 3-dimensional dynamic reconstruction for the virtual surgical planning (VSP) we performed before the surgery (Figure 11). The diagnosis was bilateral CPM hyperplasia, determining a bilateral JD. The patient was scheduled for a bilateral endoscopically assisted intraoral coronoidectomy under general anesthesia with awake fiberoptic intubation. Fibrotic shoots were detected at the pseudojoint between the CPMs and the zygoma. The entire CPM was then removed. At the end of the surgery, the interincisal distance was 50 mm with a mechanical forced opening (Figure 12). The resected specimens showed cortical bone tissue with a regular structure and orientation of the bone lamellae, determining a bilateral JD due to a true CPM hyperplasia. Articular physiotherapy was started one week after surgery using TheraBite jaw motion rehabilitation system ^TM^ (Atos medical, Padua, Italy). At 1 month after the surgery, the MMO was 25 mm and increasable after forcing, 2 months later it was 30 mm and 35 mm at 3 months. At 14 months follow-up, the patient MMO was stable at 37 mm, and the radiographic evaluation was negative for recurrence.

## 4. Results and Discussion

JD is a rare condition in which an elongated CPM interferes with the inner surface of the zygomatic arch, establishing a pseudojoint. In our review, which represents, possibly, the widest review in the literature, we report a mean age of 28.7 years old (5–73), with a difference in the age of incidence of almost 10 years between the two genders (25.7 years-old for males and 35.4 years old for females). In 12 cases, it was not possible to trace back the age of diagnosis of JD. We collected the data of 71 males (61.2%) and 34 females (29.3%). Unfortunately, 11 patients’ genders were not reported. These data are in agreement with what was previously reported in the literature, as affecting mainly men between the second and fourth decade [6]. An overall prevalence of 0.5% has also been reported, but this data could be underestimated because the onset of symptoms, such as limited mouth opening, represents just the endpoint of a longer-term development [55]. The left side is reported to be the most affected [7], which is confirmed by our analysis, in which we identified 38 cases of right JD (32.8%), 50 of the left side (43.1%) and 21 bilateral cases (18.1%). For seven cases, we were unable to retrieve information about the side of the defect. As mentioned before, JD usually remains relatively asymptomatic in its first stages, with most patients reporting only a sensation of tension during chewing. Then, it usually evolves with the limitation of mandibular movements and worsening reduction in MMO. In the reviewed cases, the mean MMO before surgical treatment was 13.9 mm (2–51). Sometimes, a distortion in the zygomatic arch projection on the affected side or a palpable moving hard mass perceptible during mandibular movements are present, particularly if JD is caused by an osteochondroma [94]. In unilateral cases, deviation to the affected side during mouth opening can be seen [75]. Pain or paresthesia are not often reported. This insidious clinical onset may be confused with several disorders such as TMD mainly, trauma arthrosis and various other causes of intra- and extra-articular ankylosis. Therefore, JD is also often mistreated. The etiology of the disease has already been widely debated. Some authors theorize a genetic or endocrine cause, and others suggest a role for temporalis muscle hyperactivity, trauma, TMJ disc displacement or a family predisposition, but it is mostly considered idiopathic [3,52,112]. Although the cause remains unknown, some authors reported periosteal hyperactivity as a trigger for ectopic metaplastic cartilage formation [5]. As stated before, according to the different amounts and patterns of bone and cartilage tissues present, the abnormally enlarged CPM can be histologically diagnosed as OC, CCE, CH, benign tumors (such as osteoma or chondroma) or other developmental anomalies [4,7,10,14,70]. We found 17 cases of CCE (14.7%), 10 cases of CH (8.6%) and 76 cases of OC (65.5%), while in 13 cases (11.2%), it was not possible to establish a definite histological diagnosis. This marked prevalence of OC as a cause of JD may partly explain the confusion over its definition and its almost exclusive bi-directional association with pathology reported by various authors. It is also interesting to note how this pathology shows different clinical and radiological patterns, leading to a wide spectrum of presentations. Considering, for example, the cases we have reported, it is possible to see how they present a pattern of increasing severity from the first to the third patient. The first patient presented an early pathologic radiological pattern, with the pseudojoint not yet fully formed with an MMO of 25 mm. It should be remembered that ankylosis, in this case, extra-articular, is established when the MMO is less than 15 mm, and this is considered an incomplete ankylosis, when the MMO is less than 5 mm, the ankylosis is complete [113,114]. In contrast, the second patient showed a well-established ipsi-pseudo-articular framework and an MMO of 20 mm. In this patient, it is also interesting to notice how the contralateral CPM was also slightly longer than normal, although not yet pathological. The third patient, on the other hand, showed a well-established bilateral pattern with perfectly developed pseudojoints and a rapidly worsening 20 mm MMO. Our experience and analysis of the literature prompted us to wonder whether it was possible to classify, according to clinical and radiological features, the vast pathological spectrum that JD expresses. We, therefore, designed a classification proposal, as shown in Table 2, in order to speculate on the possibility of assigning an index of severity and stage to the various connotations of JD. Obviously, this classification has to be intended as a proposal, considering that more cases have to be analyzed to evaluate its possible validity.

The diagnosis of JD is also often delayed because an OPG does not always permit suspecting a CPM enlargement, and the reported symptoms push the clinician to request an MRI with TMJ scans, which do not permit a proper CPM visualization. CT is frequently the last radiological examination performed. Multiplanar CT and 3D CT reconstructions, as well as VSP (Mimics, Materialise NV, Leuven, Belgium), represent the most effective tool for the correct diagnosis and surgical planning of JD cases. The definitive treatment of JD is surgical. Those submitted to surgery are patients that obviously cannot open their mouths properly, and this also entails anesthesiological issues, such as often having to resort to awake intubation or the use of a fibroscope. We saw that, despite this, in 66 cases (56.9%), the coronoidectomy was performed by an intraoral approach, while an extraoral approach was used 32 times (27.6%), and a combined approach was used 12 times (10.3%). This analysis shows slight differences in the rates reported in the literature but a significant 10% increase in the percentage of intraoral coronoidectomies previously reported [7]. Despite the difficulty of operating in such a confined space, made even more uncomfortable by the pathology, the intraoral approach is nevertheless preferred because of its relative lack of possible complications (almost no risk for facial nerve injury), such as eliminating surgical skin scars and a good possibility to reach the abnormal CPM. This approach can be implemented by various mini-invasive techniques, primarily endoscopy [115]. Other authors propose different approaches, such as coronal, hemi-coronal or trans-zygomatic [62,64]. In grade 1 cases, a conservative attitude can be taken into account, and surgery may be postponed depending on the rate of worsening of the MMO or radiological changes. In grade 2 and 3 patients, in our opinion, surgery should be considered in the first instance. We did not highlight any significant difference in MMO post-treatment, which resulted in 40.2 mm overall (20–64 mm), between patients treated via an intraoral approach (38.2 mm; 20–61 mm), extraoral (42.6 mm; 28–55 mm) and combined approaches (44.3 mm; 35–64 mm). A parameter that would be interesting to evaluate is the follow-up of these patients in order to verify any radiological or clinical recurrence. Unfortunately, in only 41 (35.3%) out of the 116 cases are the follow-up periods reported, with an average of 17.4 months. Regarding this, only two cases of recurrence (1.7%) were highlighted in our review—a percentage in line with what is reported in the literature [4]. However, it should be noticed, as it was in the case of follow-up, that no explicit mention was made about the future fate of the patients in 92 (79.3%) of the cases examined.

## 5. Conclusions

JD is a complex condition where a pseudojoint is established between an abnormal CPM and the inner aspect of the zygomatic bone, determining a progressive and worsening reduction of MMO. The diagnosis is often delayed because of its similarities with TMD and other more frequent causes of MMO reduction. CT scan that has to be performed for its provisional diagnosis, is often requested as the last radiological diagnostic examination. The spectrum of disease presentation is extremely wide, and a classification of the disease is certainly something useful that should be established internationally. Our proposal has to be intended as suggestive. A widely accepted classification may help physicians who detect this condition make a more specific diagnosis and more standardized treatment planning. The intraoral coronoidectomy approach is the most widely used procedure and allows a comparable clinical result to the extraoral approach with a lower risk of complications. More information about the follow-up of these patients is needed. A very low recurrence rate is confirmed.

## Figures and Tables

**Table 1:** Results of the literature review performed, reporting various parameters of interest that will be discussed in the discussion section.

Case No.	Author	Year	Age	Gender	Side	Type	MMO before Treatment (mm)	MMO after Treatment (mm)	Treatment	Treatment Follow-Up (Months)	Treatment Recurrence
1	Jacob [1]	1899	62	/	/	CCE	/	/	None	Not applicable	Not applicable
2	Shakleford et al. [15]	1943	15	M	L	OC	13	/	Extraoral coronoidectomy	Not reported	Not reported
3	Shakleford et al. [15]	1943	19	M	R	OC	10	/	Extraoral coronoidectomy	Not reported	Not reported
4	Brandt [16]	1943	37	F	R	CCE	25	/	Extraoral coronoidectomy	Not reported	Not reported
5	Hallam [17]	1947	18	/	/	CCE	/	/	Extraoral coronoidectomy	Not reported	Not reported
6	Shakleford et al. [18]	1949	38	M	R	OC	/	/	Extraoral coronoidectomy	Not reported	Not reported
7	Brailsford et al. [19]	1952	24	M	L	OC	13	/	Not reported	Not reported	Not reported
8	Holmes et al. [20]	1956	30	M	R/L	OC	7	/	Intraoral coronoidectomy	Not reported	Not reported
9	Holmes et al. [21]	1956	36	M	L	OC	8	/	Intraoral coronoidectomy	Not reported	Not reported
10	Ginestet et al. [22]	1956	19	/	R/L	/	/	/	Intraoral coronoidectomy	Not reported	Not reported
11	Van Ziles et al. [22]	1957	30	M	R/L	CCE	7	/	Extraoral coronoidectomy	Not reported	Not reported
12	Levine et al. [23]	1957	73	F	L	OC	6	/	Not reported	Not reported	Not reported
13	Dingman et al. [24]	1957	27	M	R	OC	8	/	Extraoral coronoidectomy	Not reported	Not reported
14	Pap et al. [25]	1958	30	M	L	OC	10	/	Extraoral coronoidectomy	Not reported	Not reported
15	Antoni et al. [26]	1958	29	M	L	OC	/	/	Intraoral coronoidectomy	Not reported	Not reported
16	Chemin et al. [27]	1958	20	/	/	/	/	/	Intraoral coronoidectomy	Not reported	Not reported
17	Shira et al. [28]	1958	14	M	R/L	/	8	/	Extraoral coronoidectomy	Not reported	Not reported
18	Lebo [29]	1961	18	M	L	OC	14	29	Extraoral coronoidectomy	Not reported	Not reported
19	Mohnac [30]	1962	18	M	R/L	OC	18	/	Extraoral coronoidectomy	Not reported	Not reported
20	Van de Vijver [31]	1962	18	/	R/L	/	/	/	Intraoral coronoidectomy	Not reported	Not reported
21	Dechaume et al. [32]	1964	13	/	/	/	/	/	Intraoral coronoidectomy	Not reported	Not reported
22	Rottke et al. [33]	1967	29	/	/	/	/	/	Intraoral coronoidectomy	Not reported	Not reported
23	Allan et al. [34]	1967	32	F	R	CCE	16	40	Intraoral coronoidectomy	Not reported	Not reported
24	Allan et al. [34]	1967	22	M	L	CCE	13	40	Intraoral coronoidectomy	Not reported	Not reported
25	Meyer [35]	1972	10	F	R	OC	20	/	Intraoral coronoidectomy	Not reported	Not reported
26	James et al. [36]	1974	52	F	R	OC	5	40	Extraoral coronoidectomy	12	No
27	Cooper et al. [37]	1974	43	F	L	OC	6	/	Intraoral coronoidectomy	Not reported	Not reported
28	Takeda et al. [38]	1975	14	F	L	OC	10	/	Extraoral coronoidectomy	Not reported	Not reported
29	Singer et al. [39]	1976	64	M	R	OC	5	/	Intra/extraoral coronoidectomy	Not reported	Not reported
30	Ramon et al. [40]	1977	45	M	R	OC	/	/	Intra/extraoral coronoidectomy	Not reported	Not reported
31	Michel et al. [41]	1977	30	F	R	/	10	/	Intraoral coronoidectomy	Not reported	Not reported
32	Norman et al. [42]	1980	21	M	L	OC	3	/	Intraoral coronoidectomy	Not reported	Not reported
33	Ito et al. [43]	1981	20	F	L	OC	3	/	Extraoral coronoidectomy	Not reported	Not reported
34	Boland et al. [44]	1983	25	/	/	/	/	/	Intraoral coronoidectomy	Not reported	Not reported
35	Ishii et al. [45]	1983	32	F	L	OC	7	41	Intraoral coronoidectomy	Not reported	Not reported
36	Ishii et al. [45]	1983	53	M	R	OC	20	37	Intra/extraoral coronoidectomy	Not reported	Not reported
37	Revington [46]	1984	24	M	R	CCE	9	/	Extraoral coronoidectomy	6	Not reported
38	Tucker et al. [47]	1984	16	M	R	CH	22	/	Intraoral coronoidectomy	Not reported	Not reported
39	Macleod [48]	1987	46	F	/	CCE	5	/	Intraoral coronoidectomy	Not reported	Not reported
40	Schwartz et al. [49]	1987	15	M	L	OC	18	/	Extraoral coronoidectomy	Not reported	Not reported
41	Halling et al. [50]	1989	22	M	R	/	21	/	Intraoral coronoidectomy	Not reported	Not reported
42	Goudot et al. [51]	1989	45	/	L	/	/	/	Intraoral coronoidectomy	Not reported	Not reported
43	Totsuka et al. [52]	1990	37	F	L	OC	2	28	Intraoral coronoidectomy	Not reported	Not reported
44	Rames et al. [53]	1990	36	/	R/L	CH	/	/	Intraoral coronoidectomy	Not reported	Not reported
45	Asanami et al. [54]	1990	17	M	L	CCE	8	64	Intra/extraoral coronoidectomy	36	Not reported
46	Honig et al. [55]	1993	22	M	R	CH	21	/	Intraoral coronoidectomy	Not reported	Not reported
47	Kerscher et al. [56]	1993	45	M	L	OC	14	32	Intraoral coronoidectomy	Not reported	Not reported
48	Smyth et al. [57]	1994	15	M	R/L	CH	4	40	Extraoral coronoidectomy	96	Yes (96 months)
49	Gibbons et al. [58]	1995	34	M	R/L	OC	19	34	Intraoral coronoidectomy	Not reported	Not reported
50	Cenetoglu et al. [59]	1996	19	M	L	OC	12	48	Intraoral coronoidectomy	12	Not reported
51	Kermer et al. [60]	1996	40	M	L	OC	/	/	Extraoral coronoidectomy	Not reported	Not reported
52	Mizumoto et al. [61]	1996	43	M	L	OC	11	49	Extraoral coronoidectomy	12	Not reported
53	Constantinides et al. [62]	1997	31	M	R	OC	10	/	Extraoral coronoidectomy	12	Not reported
54	Gross et al. [63]	1997	22	M	L	OC	20	40	Intraoral coronoidectomy	Not reported	Not reported
55	Chen et al. [64]	1998	57	F	L	OC	14	42	Extraoral coronoidectomy	72	No
56	Takenobu et al. [65]	1998	56	F	L	OC	2	30	Intraoral coronoidectomy	12	Not reported
57	Manganaro [66]	1998	26	F	L	OC	/	/	Intraoral coronoidectomy	Not reported	Not reported
58	Ishii et al. [67]	1998	25	M	R	CH	22	40	Intraoral coronoidectomy	Not reported	Not reported
59	Chicareon et al. [68]	1999	5	M	R	OC	/	/	Not reported	Not reported	Not reported
60	Hernandez-Alfaro et al. [8]	2000	22	M	L	OC	21	52	Extraoral coronoidectomy	6	Not reported
61	Escuder y de la Torre et al. [14]	2001	19	M	L	/	7	/	Intraoral coronoidectomy	Not reported	Not reported
62	Escuder y de la Torre et al. [14]	2001	16	/	R/L	OC	14	49	Intraoral coronoidectomy	12	No
63	Emekli et al. [4]	2002	26	F	R	OC	10	35	Intraoral coronoidectomy	6	No
64	Emekli et al. [4]	2002	21	M	R	OC	8	40	Intra/extraoral coronoidectomy	Not reported	Not reported
65	Roychoudhury et al. [6]	2002	32	M	L	OC	0	39	Extraoral coronoidectomy	>12	Not reported
66	Capote et al. [11]	2005	23	F	L	CCE	30	40	Intraoral coronoidectomy	12	Not reported
67	Matsumoto et al. [69]	2005	38	M	L	OC	12	41	Intra/extraoral coronoidectomy	3	Not reported
68	Akan et al. [70]	2006	24	M	R/L	CCE	15	30	Intraoral coronoidectomy	Not reported	Not reported
69	Villanueva et al. [71]	2006	44	F	L	OC	30	43	Intraoral coronoidectomy	Not reported	Not reported
70	Dede et al. [72]	2007	20	M	R/L	OC	6	/	Intraoral coronoidectomy	Not reported	Not reported
71	Zhong et al. [73]	2009	39	F	R	OC	8	31	Intraoral coronoidectomy	9	Not reported
72	Thota et al. [74]	2009	15	M	R/L	CCE	15	44	Intraoral coronoidectomy	14	No
73	Osman et al. [75]	2009	43	F	R	OC	14	30	Intraoral coronoidectomy	6	Not reported
74	D’Ambrosio et al. [13]	2009	39	M	L	OC	/	/	Intraoral coronoidectomy	Not reported	Not reported
75	Yesildag et al. [76]	2010	16	M	R	OC	12	52	Extraoral coronoidectomy	14	Not reported
76	Takafuji et al. [77]	2011	21	M	R	OC	20	48	Intraoral coronoidectomy	Not reported	Not reported
77	Ajila et al. [78]	2011	28	M	L	OC	7	/	Intraoral coronoidectomy	12	No
78	Coll-Anglada et al. [12]	2011	52	F	R	OC	8	41	Intraoral coronoidectomy	6	Not reported
79	Acosta-Feria et al. [79]	2011	55	F	R	OC	20	40	Extraoral coronoidectomy	24	No
80	Sreeramaneni et al. [7]	2011	45	F	L	OC	5	40	Intra/extraoral coronoidectomy	3	Not reported
81	Aoki et al. [80]	2012	18	M	R	OC	51	61	Intraoral coronoidectomy	15	No
82	Pacheco Ruiz et al. [81]	2012	28	M	R/L	OC	2	40	Intra/extraoral coronoidectomy	3	Not reported
83	Tavassol et al. [82]	2012	13	M	R/L	CH	10	48	Intraoral coronoidectomy	Not reported	Not reported
84	Choi et al. [83]	2013	13	M	R/L	CCE	15	40	Intraoral coronoidectomy	4	Not reported
85	Choi et al. [83]	2013	13	M	R/L	CCE	20	45	Intraoral coronoidectomy	Not reported	Yes (36 months)
86	Stringer et al. [84]	2013	27	M	L	OC	10	40	Intraoral coronoidectomy	Not reported	Not reported
87	Hosein et al. [85]	2013	15	M	L	OC	22	41	Intraoral coronoidectomy	18	Not reported
88	Losa-Munoz et al. [86]	2014	42	M	R	OC	20	41	Intraoral coronoidectomy	Not reported	Not reported
89	Dandriyal et al. [87]	2014	20	F	L	OC	15	40	Intraoral coronoidectomy	54	No
90	Zarembinski et al. [88]	2014	50	M	R	/	35	/	None	Not reported	Not reported
91	Fan et al. [89]	2014	20	M	L	OC	25	45	Intra/extraoral coronoidectomy	20	No
92	Rahim et al. [90]	2014	19	M	L	OC	22	/	Not reported	Not reported	Not reported
93	Reddy et al. [91]	2014	21	F	R	OC	12	42	Extraoral coronoidectomy	Not reported	Not reported
94	Sinha et al. [92]	2014	58	M	L	OC	/	/	Intraoral coronoidectomy	Not reported	Not reported
95	Sawada et al. [93]	2015	14	M	L	OC	10	20	Intraoral coronoidectomy	Not reported	No
96	Shin et al. [94]	2016	39	F	L	/	21	41	Intra/extraoral coronoidectomy	36	No
97	Mohanty et al. [95]	2016	18	M	R	OC	11	40	Extraoral coronoidectomy	36	No
98	Robiony et al. [96]	2016	59	F	R	OC	9	49	Extraoral coronoidectomy	6	Not reported
99	Imen et al. [97]	2016	29	M	L	OC	5	55	Extraoral coronoidectomy	Not reported	Not reported
100	Gangoli et al. [98]	2017	15	F	L	OC	9	34	Intraoral coronoidectomy	Not reported	Not reported
101	Choontharu et al. [99]	2018	16	F	L	OC	43	/	Intraoral coronoidectomy	6	No
102	Roscher et al. [100]	2018	18	F	L	OC	20	41	Intraoral coronoidectomy	12	Not reported
103	Villegas Cisneros et al. [101]	2018	10	M	R/L	CH	19	35	Intra/extraoral coronoidectomy	Not reported	Not reported
104	Lan et al. [102]	2019	34	F	R	OC	5	36	Intraoral coronoidectomy	21	No
105	Kono et al. [103]	2019	51	M	L	OC	20	42	Intraoral coronoidectomy	30	No
106	Samandari et al. [104]	2019	28	M	L	CH	25	/	Intraoral coronoidectomy	Not reported	Not reported
107	Gomez et al. [105]	2020	54	F	L	OC	12	22	Intraoral coronoidectomy	12	Not reported
108	Jimenez Alvarez et al. [106]	2020	15	M	R	OC	10	40	Intraoral coronoidectomy	72	Not reported
109	Avelar et al. [107]	2020	6	M	R/L	CH	10	28	Extraoral coronoidectomy	Not reported	No
110	Okazawa et al. [108]	2020	27	M	L	OC	4	60	Intra/extraoral coronoidectomy	12	No
111	Khadembaschi et al. [109]	2020	14	M	R/L	CCE	8	45	Extraoral coronoidectomy	18	Not reported
112	Alam et al. [110]	2021	32	M	R	OC	8	38	Extraoral coronoidectomy	Not reported	Not reported
113	Leal et al. [111]	2021	11	F	L	CCE	20	25	Intraoral coronoidectomy	Not reported	Not reported
114	Raccampo et al.	2022	19	F	R	CCE	25	40	Intraoral coronoidectomy	12	No
115	Raccampo et al.	2022	18	M	R	OC	20	44	Intraoral coronoidectomy	12	No
116	Raccampo et al.	2022	23	M	R/L	CH	20	37	Intraoral coronoidectomy	14	No

**Table 2:** Our proposal of JD’s classification.

Classification Proposal		
**Grade 1**	Grade 1 A	An ipsilateral formation of a pseudojoint between an abnormal CPM and the inner aspect of the zygomatic bone determining an extra-articular ankylosis with a MMO ≥ 20 mm.
	Grade 1 B	A bilateral formation of a pseudojoint between an abnormal CPM and the inner aspect of the zygomatic bone determining an extra-articular ankylosis with a MMO ≥ 20 mm.
**Grade 2**	Grade 2 A	An ipsilateral formation of a pseudojoint between an abnormal CPM and the inner aspect of the zygomatic bone determining an extra-articular ankylosis with a 20 ≤ MMO ≥ 10 mm.
	Grade 2 B	A bilateral formation of a pseudojoint between an abnormal CPM and the inner aspect of the zygomatic bone determining an extra-articular ankylosis with a 20 ≤ MMO ≥ 10 mm.
**Grade 3**	Grade 3 A	An ipsilateral formation of a pseudojoint between an abnormal CPM and the inner aspect of the zygomatic bone determining an extra-articular ankylosis with a MMO ≤ 10 mm.
	Grade 3 B	A bilateral formation of a pseudojoint between an abnormal CPM and the inner aspect of the zygomatic bone determining an extra-articular ankylosis with a MMO ≤ 10 mm.

## Data Availability

The data presented in this study are available on request from the corresponding author. The data are not publicly available due to privacy restrictions.
